# Acute lymphoblastic leukemia subsequent to temozolomide use in a 26-year-old man: a case report

**DOI:** 10.1186/1752-1947-4-274

**Published:** 2010-08-18

**Authors:** Asim Jamal Shaikh, Nehal Masood

**Affiliations:** 1 Section of Medical Oncology, The Aga Khan University Hospital, Karachi, Pakistan

## Abstract

**Introduction:**

We report the development of acute lymphoblastic leukemia in a patient in whom temozolomide was used for the treatment of a brain tumor. Unlike that of other alkylating agents, the leukemogenic potential of temozolomide is considered to be very low, and very rarely are such cases reported.

**Case Presentation:**

A 26-year-old Pakistani man who was treated for glioblastoma with temozolomide in an adjuvant setting was diagnosed to have acute lymphoblastic leukemia one year after stopping temozolomide.

**Conclusion:**

Temozolomide is a highly active agent, used in the management of high-grade brain neoplasms. The agent is generally regarded to be safe, with an acceptable safety profile. Very few cases of myelodysplasia associated with temozolomide use have been reported. We report here the first case of acute lymphoblastic leukemia, which developed in a young man about one year after he finished taking temozolomide. This should provide further insight into a possible toxicity profile of this alkylating agent. This finding should be of interest to physicians in general and to medical oncologists in particular.

## Introduction

Survival rates from aggressive, relapsed, refractory, or high-grade brain tumors are generally poor, with the median survival for some being less than one year [1]. With increased survival, however, the long-term toxicities of the available chemotherapeutic agents used in aggressive brain cancers have become more prominent [2]. Alkylating agents remain the most active agents known for the treatment of aggressive and high-grade brain neoplasms. Treatment-related myelodysplasia (t-MDS) and acute leukemia (t-AL) have remained a concern of prolonged exposure to alkylating agents [3]. Temozolomide (TMZ) is an oral second-generation alkylating agent with activity against recurrent high-grade gliomas and has been considered efficacious and relatively safe [4]. Here we report a case of t-ALL in a patient who received TMZ for the treatment of high-grade mixed glioma.

## Case Report

A 26-year-old Pakistani man presented with history of new-onset seizures. Magnetic resonance imaging (MRI) of the brain revealed a contrast-enhancing lesion in the right frontoparietal region with compressions and a shift of the midline. The mass was resected in August 2007 and confirmed to be a mixed glioma with components of both astrocytoma and oligodendroglioma, WHO grade II. About six weeks after surgery, the patient was brought back with a new history of seizures. An MRI examination revealed a gross local recurrence at the site of the previous surgery, which was infiltrating within the sulci of the brain matter. Based on the clinical behavior and surgical unresectability of the tumor, he was treated with concurrent chemoradiation therapy (radiation: 6000 cGY/temozolomide, 75 mg/m^2^). He showed an excellent response to concurrent chemoradiotherapy, with a complete disappearance of the recurred lesion. He was given a total of six cycles of TMZ (150 mg/m^2^, days one to five, every 28 days). He completed chemotherapy in January 2008 and remained well, without evidence of recurrence, on surveillance MRI scans. He recently came in complaining of easy bruisability; blood counts revealed an elevated white blood cell count (total leukocyte count; 20,000 per deciliter; 16% neutrophils; 78% lymphocytes) and thrombocytopenia (platelet count, 16,000 per deciliter). Bone-marrow aspirate revealed diffuse infiltration with blast cells consistent with acute leukemia. Peripheral blood flow cytometry on immunophenotyping with five-color cytomics (fc500 Beckman Coulter flow cytometer) showed this population of cells with bright reactivity with Pan-T-markers (that is, CD5, CD7, and cytoplasm cCD3, along with CD45). Positivity of this population with Tdt was also very prominent, so immunophenotypic results were consistent with precursor-T-acute lymphoblastic leukemia (Pre-T-ALL). Bone marrow cytogenetics revealed a normal karyotype and negative Philadelphia chromosome. He is currently undergoing treatment.

## Discussion

We report, to the best of our best knowledge and search of the literature, what appears to be the first reported case of Philadelphia-negative true ALL developing subsequent to the use of TMZ. Some case reports exist of myelodyplasia rapidly transforming in undifferentiated leukemia [3,5] and one report of Ph negative T-ALL in a patient receiving treatment [6].

TMZ is an oral alkylating agent that is now known to be active against a variety of CNS neoplasms. After oral absorption, it spontaneously hydrolyzes to methyltriazen-1-yl imidazole-4-carboxamide (MTIC). MTIC degrades to a highly reactive cation that methylates guanines in DNA at the O6 position, causing base-pair mismatch. Unsuccessful cycles of mismatch repair eventually lead to breaks and permanent nicks in the daughter strand, preventing mitotic division, and the cell undergoes apoptosis [7,8]. The action of TMZ has been shown to be augmented in the concurrent presence of radiation, so the proof of efficacy and superiority of TMZ has led to a paradigm shift in the management of aggressive CNS gliomas [1]. Although the recommended treatment-cycle length is six months after initial treatment, with concurrent chemoradiotherapy, some neuro-oncologists prefer to use it indefinitely [9]. A recent survey of physicians who used TMZ for more than one year, on average, found it to be completely safe, except for grade II and III myelosuppression [10]. All alkylating agents are considered to carry a five to ten percent mutagenic risk potential for development of myeloid leukemia, but not for lymphoblastic leukemia. TMZ is a new alkylating agent; its safety profile and lack of data on any mutagenic potential has led to its incorporation in a large number of studies, for the range from malignant gliomas to malignant melanomas [11]. Little consistent data exist regarding the toxicity of TMZ, so questions have been raised about its mutagenic potential. Some clinical trials have started to include carcinogenic potential as a point of assessment in long-term safety monitoring of the drug [11]. Hartmut Geiger *et al*. [12] published data that reveal the mutagenic potential of TMZ for bone marrow cells *in vivo *in the mouse model system.

## Conclusion

TMZ has unequivocally shown its therapeutic potential in randomized clinical trials as an effective, relatively safe, and generally well-tolerated therapy for aggressive CNS neoplasms, resulting in better overall survival. Because it is a relatively new and unique alkylating agent, the short-term and long-term data regarding safety, especially leukemogenic potential, must have further time to mature. Although the association is unlikely to be a random finding, the association between TMZ and treatment-related leukemia deserves further study.

## Consent

Written informed consent was obtained from the patient for publication of this case report and accompanying images. A copy of the written consent is available for review by the Editor-in-Chief of this journal.

## Conflict of Interest

Both authors declare no conflict of interest with reference to material published.

## Authors' contributions

AJS wrote the manuscript, searched the literature, and aided in patient coordination. NM wrote the manuscript and searched the literature. Both authors read and approved the final manuscript.
